# Effect of Depth of Electroacupuncture on the IPSS of Patients with Benign Prostatic Hyperplasia

**DOI:** 10.1155/2019/1439141

**Published:** 2019-12-12

**Authors:** Hongwei Yuan, Na Wei, Yanfeng Li, Lifeng Yu, Yunshu Zhang, Wei Lin Ong, Ni Bai, Mengxi Wang, Yuting Fu, Yunxia Liu

**Affiliations:** ^1^Department of Acupuncture and Moxibustion, Dongzhimen Hospital Affiliated to Beijing University of Chinese Medicine, Beijing 100700, China; ^2^Department of Function, Dongzhimen Hospital Affiliated to Beijing University of Chinese Medicine, Beijing 100700, China; ^3^Department of Urology, Dongzhimen Hospital Affiliated to Beijing University of Chinese Medicine, Beijing 100700, China

## Abstract

This study aimed to evaluate the efficacy of electroacupuncture (EA) on Ciliao (BL 32) and Zhongliao (BL 33) acupoints at different depths for the treatment of benign prostatic hyperplasia (BPH) through a single-blind randomized controlled trial. All 120 patients diagnosed with BPH were randomly allocated to an experimental group (deep insertion group, DI group, *n* = 60) and control group (shallow insertion group, SI group, *n* = 60) 3 times a week for 4 weeks. The observed results included the International Prostate Symptom Score (IPSS), Quality of Life score (QOL), maximum urine flow rate (Qmax), and postvoid residual urine volume (PVR). After treatment, at both depths, the BPH symptoms of patients were improved by EA. There were significant differences between the IPSS, QOL, and the effective rate of the experimental group and the control group (*P* < 0.05). Although the observed PVR and Qmax were better than those before treatment, there was no statistical significance between two groups (*P* > 0.05). Thus, EA with deep insertion can effectively improve the patients' urinary symptoms and quality of life, and it will be suitable for clinical application.

## 1. Introduction

Benign prostatic hyperplasia (BPH) is one of the common urinary system diseases in the middle-aged and elderly males [1]. The lower urinary tract symptoms (LUTS) are the main clinical manifestations, including bladder irritation symptoms such as urinary frequency, urgency, and nocturia and urinary obstruction symptoms such as prolonged urination time, intermittent flow, and progressive dysuria. There is a positive correlation of the occurrence of BPH and age. It often occurs in males after the age of 40, with more than 50% occurrence rate at the age of 60 and as high as 83% after the age of 80 [2, 3]. In addition, the symptoms of dysuria become more severe in older patients, which has a negative impact on patients' quality of life. Currently, treatments of BPH include usage of drugs, surgical resection or minimally invasive intervention. The drugs used include alpha blockers (ABs), 5-alpha reductase inhibitors (5ARI), or combination of multiple drugs [4]. In recent years, acupuncture is an increasing popular method in the treatment of diseases. Similarly, in the treatment of BPH, acupuncture is also proven to be effective in alleviating the bladder irritation symptoms [5–7]. Also, to improve the efficacy of acupuncture treatment, the usage of frequency electric stimulation with tradition acupuncture, electroacupuncture (EA), is also introduced to enhance the “Deqi” sensation of acupuncture [8]. However, there is no concrete study on the effect of acupuncture depth on the efficacy of the acupuncture treatment.

The aim of study was to observe EA in the treatment of BPH to assess the correlation between acupuncture depth and clinical efficacy through a randomized single-blind controlled study.

## 2. Materials and Methods

### 2.1. Study Design

**Figure 1:**
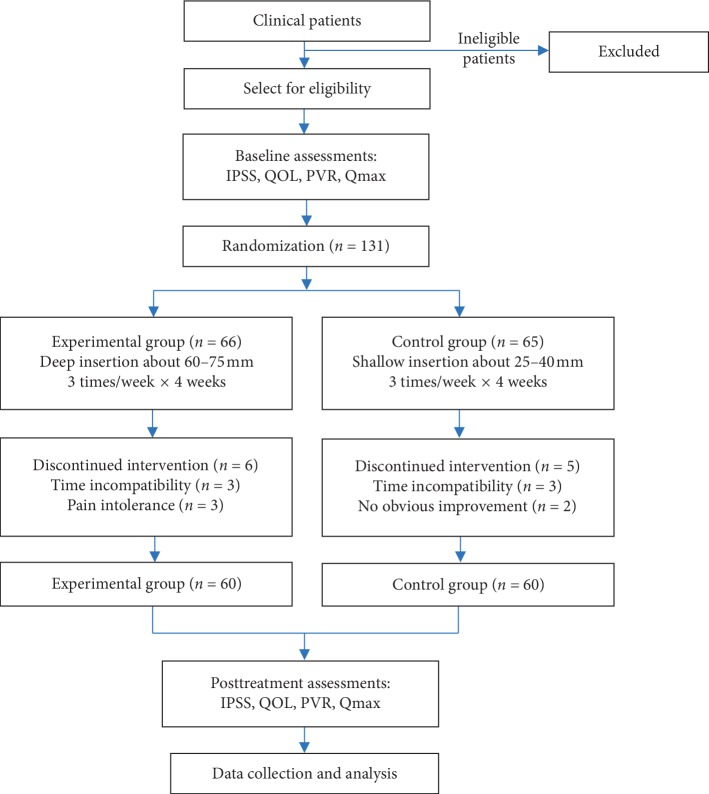
Flowchart of the study.

This study is a single-blind randomized controlled trial to determine the effect of acupuncture depth on the efficacy of EA treatment for BPH at Ciliao (BL 32) and Zhongliao (BL 33) points. All participants of the study were recruited from the outpatient clinic of the Dongzhimen Hospital from Dec 2017 to Apr 2019. Participants were all diagnosed with BPH according to the diagnostic standard and degree of standards for diagnosis and curative effect of the Chinese Urological Association's Diagnostic and Therapeutic Guidelines (2014) [9]. In the recruitment of patients, the possibility of BPH was considered as the first priority for male patients over 50 years of age with LUTS as the main complaint, and detailed medical history was inquired for initial evaluation. The diagnosis was then confirmed after a series of specialized physical, laboratory, and imaging examinations. The flowchart of the study designed is represented in Figure 1.

The sample size was estimated from a previous study that evaluated IPSS differences between two groups as its primary outcome [10]. A mean IPSS difference of 2.9 (*S* = 2.9) between the two groups and 1.6 (*δ* = 1.6) between post-treatment of two groups were used to calculate the sample size in this study (*α* = 0.05, *β* = 0.2).

(1) $$\begin{array}{c} n1=n2=2\times {\left(t_{\alpha }+t_{\beta }\right)}^{2}\times {\left(\frac{S}{\delta }\right)}^{2}. \end{array}$$

A total of 41 subjects were required in each group; therefore, 60 patients would be sufficient in our study. Throughout the clinical study, data obtained from 120 participants were included in the analysis (DI group, *n* = 60; SI group, *n* = 60).

A researcher who was not involved in the treatment and data analysis handed out the serial number and opaque, sealed envelopes to patients. They were randomly allocated to receive the experimental group (deep insertion group, DI group) or control group (shallow insertion group, SI group) based on the odd (DI group) or even (SI group) numbers assigned in the envelope. All patients in both groups received EA treatment 3 times a week over a period of 4 weeks. In order to maintain blinding through this clinical trial, the patients, statisticians, and the data evaluators were blind to the allocation of the groups. The allocation of the groups are known to the doctors performing as it is not feasible to conceal allocation. Apart from the varied needle insertion depth, all other parameters of the trial were controlled. During the intervention period, acupuncturists and data collection staff would visit patients at different time intervals to avoid exchange of information.

The study protocol was approved by Dongzhimen Hospital Affiliated to Beijing University of Chinese Medicine Medical Ethics Committee (approval no. DZMEC-KY-2017-49) and registered on the Clinical Trials. The study conformed to the ethical guidelines of the Declaration of Helsinki. All the patients consented to participate in the trial and provided written consent form.

### 2.2. Diagnostic Criteria

According to the diagnostic standard and degree of standards for diagnosis and curative effect of the Chinese Urological Association's Diagnostic and Therapeutic Guidelines (2014) [9], the diagnostic criteria are as follows: (a) urinary symptoms: frequency, urgency, incontinence, nocturia, dysuria, intermittent flow, and incomplete emptying of the bladder; (b) digital rectal examination (DRE): assessment of anal sphincter muscle tension and size of the prostate, texture; (c) prostate ultrasonography: Prostate gland hypertrophy and residual urine volume. (d) uroflowmetry: maximum urine flow rate <15 ml/s at urine volume of more than 150 ml.

### 2.3. Inclusion Criteria

(a) Aged 50–80 years old; (b) diagnosed with BPH for more than 3 months; (c) abstain from medication for BPH for more than 2 weeks before the start of treatment; (d) IPSS within moderate and severe range (8–19 for moderate, 20–35 for severe); (e) stable vitals; and (f) voluntarily participation of the study.

### 2.4. Exclusion Criteria

(a) Patients receiving other treatment for BPH; (b) patients with various urinary tract infections; (c) patients with other diseases affecting urination; (d) patients with severe diseases including but not limited to cardiovascular, cerebral, hepatic, renal, or hemopoietic diseases; (e) patients with mental disorder; (f) patients with unstable vitals; and (g) patients with phobia of acupuncture.

### 2.5. Interventions

The acupuncture treatment prescription was developed in a consensus process by experienced acupuncturists. The clinical trial was performed by a group of five qualified acupuncturists with Chinese medicine practitioner license from the Ministry of Health of the People's Republic of China. All the acupoints selected in the study were according to the People's Republic of China names and locations of acupoints (GB/T12346-2006). In both groups, patients were treated with acupuncture on bilateral BL 32 and BL 33.

Experimental group (DI group): 0.30 mm × 75 mm sterile disposable needles (Beijing Zhongyantaihe Medical Instruments Co, Ltd., China) were used in the DI group. Patients should empty their bladder prior to each session. The acupuncturist requests the patients to maintain a prone position, and disinfect the local area before the insertion of needles. The needles would then be inserted obliquely at a depth of 60–75 mm, through lifting and thrusting the needles as well as twirling and rotating the needles until the patient obtain the “Deqi” sensation. This needle sensation is often described as soreness or a dull ache. The sensation of the needle should be felt as a continuous transmission to the anterior pudendal area. In addition, a continuous electrical wave was delivered via a G6805-II electric stimulator (Shanghai Huayi Medical Instrument Co, Ltd., China) to the acupoints at 20 Hz frequency for 30 min during the treatment. The current intensity used was determined by patients' tolerance. Patients were treated 3 times per week for a total of 4 weeks.

Control group (SI group): 0.25 mm × 40 mm sterile disposable needles (Beijing Zhongyantaihe Medical Instruments Co, Ltd.) were used in the SI group, and acupuncturists manipulate the needles to make patients feel needling sensation. Besides the insertion depth at 25–40 mm, all other parameters used in the SI group are controlled.

### 2.6. Observed Measurements

#### 2.6.1. The Primarily Observed Measurement

The International Prostate Symptom Score (IPSS) is the primarily observed measurement in this experiment. IPSS is a subjective assessment of patients with BPH micturition symptom severity and is considered to be one of the questionnaires for evaluating BPH [11]. This assessment contains 7 factors including incomplete emptying, frequency, interruption, urgency, weak stream, hesitancy, and nocturia. Each factor has a grading score of 0–5, and the cumulative score of the factors is the total IPSS of the patient. The total IPSS range from 0–35. The score represents the severity of BPH, and the higher the IPSS, the more severe the manifestation of the disease seen in the patient. The results were divided into 3 grades, namely, mild, moderate, and severe BPH based on the scores (1–7 for mild, 8–19 for moderate, and 20–35 for severe).

#### 2.6.2. The Secondary Observed Measurements

The secondary observed measurements of the experiment are the Quality of life score (QOL), Maximum urine flow rate (Qmax), and Postvoid residual urine volume (PVR). QOL is an essential indicator for evaluation of distress caused by BPH [12], the degree of disturbance of LUTS in the daily lives of BPH patients, also known as distress score. It has a grading range of 0–6, and the lower the score, the better the quality of life. Although both IPSS and QOL are relatively subjective as it is based on the individual patients' feelings of their symptoms, it allows doctors to better understand the severity of the patients' BPH. Qmax refers to the maximum amount of urine discharged through the urethral opening in a unit of time, objectively evaluating the micturition of patients. PVR is a reflection of the urination function of the bladder to certain extent, and it is used as one of the important indicators for the diagnosis of BPH. Studies have shown that Qmax and PVR indicators have clinical significance in determining the degree of urination difficulty of BPH patients [13]. The International Continence Society (ICS) recommends to perform the testing of PVR immediately after inspection of urine flow rate, as the combination of the two tests can further improve the diagnosis rate of urination disorder [14].

#### 2.6.3. Measurement of Efficacy

1. *Highly Effective*. Significant improvement in the patient's BPH symptoms, with the IPSS score decreased by more than 2/3
2. *Effective*. Slight improvement in the patient's BPH symptoms, with the IPSS score decreased by 1/3 to 2/3
3. *Ineffective*. No improvement in the patient's BPH symptoms, with the IPSS score decreased by less than 1/3

### 2.7. Statistical Analysis

All statistical analyses were performed by SPSS version 25.0 (SPSS Inc. Armonk, NY: IBM Corp.). The Shapiro–Wilk W test was used to detect if the data fitted normal distribution in two groups. The data were normally distributed and presented as mean ± standard deviation ($\bar{x}$ ± *s*). Otherwise, the data were presented as median (interquartile range). The pre-post scores of IPSS of each group were respectively analyzed by nonpaired *T*-test while the scores between the two groups were analyzed by paired *T*-test. QOL, PVR, and Qmax were analyzed between the two groups using the Mann–Whitney *U*-test. The total effective rate was analyzed by Pearson's Chi-Square test. All reported *P* values were two sided, and *P* < 0.05 was considered statistically significant.

## 3. Results

BPH patients fulfilling the inclusion criteria were randomly divided into two groups. Prior to treatment, statistical analysis of the age, course of disease, and degree of disease of the patients in both groups were performed. There is no statistical difference in the two groups (*P* > 0.05). The characteristics of the patients is reflected in Table 1.

In the DI group, there were significant differences in the total IPSS and urinary symptoms score before and after the treatment (*P* < 0.05; Table 2). In the SI group, there was a significant difference in the total IPSS before and after the treatment (*P* < 0.05; Table 2). There were no significant differences in the interruption and urgency symptom scores before and after the treatment (*P* > 0.05; Table 2). With the exception of interruption and urgency symptoms score, there were significant differences in the other urinary symptoms scores before and after the treatment. From the results, it can be concluded that EA treatment of BL 32 and BL 33 acupoints can effectively improve the patients' clinical symptoms of BPH. However, by analysis of the pre-post-change scores, there was a greater improvement of total IPSS in the DI group compared with that in SI group, especially in terms of frequency, urgency, and nocturia symptoms (*P* < 0.05; Table 3). There were no significant differences in the incomplete emptying, interruption, weak stream, and hesitancy scores before and after the treatment between two groups (*P* > 0.05; Table 3).

Prior to the treatment, there were no significant differences in QOL, PVR, and Qmax between the two groups (*P* > 0.05; Table 1). In the DI group, there were significant differences in QOL and PVR before and after the treatment (*P* > 0.05; Table 4). Although there was an improvement in Qmax, there were no statistical differences before and after the treatment (*P* > 0.05; Table 4). In the SI group, there were significant differences in QOL, PVR, and Qmax before and after the treatment (*P* < 0.05; Table 4). In terms of the pre-post-change score of QOL, there was a significant difference between the DI group and SI group (*P* < 0.05; Table 5). Hence, the DI group was better at improving the QOL score compared with the SI group. As a result, it can be concluded that deep insertion electroacupuncture treatment of BL 32 and BL 33 acupoints was more effective in improving the quality of life of BPH patients (Table 5).

At the end of treatment, the total effective rate of the DI group was 68.33%, among which there were 15 highly effective cases, 26 effective cases, and 19 ineffective cases. The total effective rate of the SI group was 38.33%, among which there were 1 highly effective case, 22 effective cases, and 37 ineffective cases. There was a significant difference between the effective rates of the DI group and SI group (*P* < 0.001; Table 6). From the results, there was a higher effective rate in the DI group than SI group.

## 4. Discussion

The main clinical manifestation of BPH is difficulty in micturition. It is classified under *Longbi* in Traditional Chinese Medicine (TCM) [15] and is often observed in males aged over 50. According to the TCM theory, the occurrence of the disease is related to the disturbance of the bladder and kidney, and also the spleen, liver, and lung [16]. BPH is a common disease in the aging male, as with age, deficiency of kidney Qi is more apparent. The gasify function of the bladder is affected, and hence there is a retention of urine in the bladder, causing the dribble and shortness in urination and obstructed micturition as the disease progresses.

Acupuncture is an effective method in the treatment of urinary disturbance diseases. The points BL 32 and BL 33 adopted in this study conformed to safety and effective acupoints selection criteria [17–19]. From the perspective of meridians and collaterals theory, these points both belong to the bladder meridian, both located in the posterior sacral pore of the lumbagosacral. According to the Classic of Acupuncture and Moxibustion, BL 32 is located at the second empty place, and BL 33 is located at the third empty space, both followed by Jiaji (EX-B2). Needle sensation directed to the disease location can stimulate bladder Qi, promoting bladder gasification, with the effect of promoting diuresis and relieving stranguria. According to the theory that meridians and collaterals could treat the diseases where they circulate, stimulation of these acupoints mainly treat diseases of the genitourinary system. From the perspective of neuroanatomy, these acupoints are located at the second and third posterior sacral, where the sacral nerves S2 and S3 pass through, respectively. By using acupuncture to stimulate the sacral plexus, the needle sensation will transmit to the anus, perineum, and the lower abdomen [20]. It will also stimulate the sacral micturition center, hence affecting the bladder detrusor and urethral sphincter, thus improving the bladder irritation and symptoms of urinary tract obstruction. In addition, the continuous low-frequency pulse of EA can enhance the effect of acupuncture to a certain extent [21]. This method is minimally invasive, simple to perform, economical, and safe, hence a widely accepted treatment method among BPH patients.

Previous studies have confirmed the clinical efficacy of EA in the treatment of BPH had better effects than positive drugs [22, 23]. A study comparing the effects of acupuncture and sham acupuncture on BPH showed that acupuncture intervention significantly improved IPSS in patients with BPH in the short term (4–6 weeks). There was no significant difference in long-term efficacy (12–18 weeks), [6]. Animal experiments showed that acupuncture with deep insertion was better than regular insertion to treat urinary retention after spinal cord injury [24]. There is insufficient evidence that the depth of needle insertion during acupuncture treatment affects the clinical efficacy of the treatment of BPH. Hence, this study aims to assess the correlation between acupuncture depth and clinical efficacy of the treatment of BPH via EA.

This study found that regardless of needle depth, EA performed on BL 32 and BL 33 acupoints were effective in improving the symptoms of BPH. In the DI group, 0.30 mm × 75 mm needles were used to penetrate the posterior sacral foramen, stimulating the sacral plexus directly. In the SI group, 0.25 mm × 40 mm needles were used, with the main purpose of producing needle sensation locally in the affected area. Studies have shown that the degree of prostatic hyperplasia is positively correlated with IPSS [25]. According to our statistical results, at both insertion depths, significant improvement in the IPSS and QOL scores are observed, with the experimental group having a greater improvement than the control group. However, no obvious improvement in the Qmax score is observed in both groups. According to our analysis, the most probable reason for the lack of improvement is the limited treatment period of the study. With this limited time period, improvement in the regulation of functions can be observed, but changes in the physical lesions is not significant. According to the TCM theory of Qi extending affected parts, acupuncture improves the circulation of Qi to the affected areas, improving the symptoms and hence obtaining clinical efficacy. In the experimental group, as deep insertion of the needle allows the acupuncture sensation to reach the “site” of disease directly, more prominent effect can be observed, and the treatment can be easily repeated. In conventional acupuncture, it is necessary to ensure the Deqi sensation, transmitting the needle sensation to the “site” of disease via methods of twisting and turning the needle. In conclusion, EA on BL 32 and BL 33 to Qi extending affected parts is the key factor in the treatment of BPH via acupuncture. The selection of acupuncture depth should be based on comprehensive consideration including the basic physical condition of patients, the thickness of lumbosacral fat, and the tolerance to therapeutic stimulation amount.

### 4.1. Limitations

The present study had some limitations: (a) according to previous studies that have confirmed the clinical efficacy of EA in treating BPH, the study only compared the effects of different acupuncture depths on symptoms and lacked control with positive drugs and sham acupuncture. (b) This study evaluated the efficacies of pre and post treatments and is statistically significant, but the long-term effect of EA has not been evaluated yet. Our team will continue to follow-up in order to obtain more clinical data and clarify the continuous effect of EA treatment (3 times/week × 4 weeks) and explore the dose-effect relationship of acupuncture. (c) It is speculated that the deep acupuncture depth may be more conducive to improving symptoms than the shallow acupuncture from our small sample study, and we will need large-scale and multicenter studies to further verify the therapeutic effect of acupuncture depth on BPH, to promote the clinical application of EA in treating BPH, and hence develop a more effective and standardized treatment regimens for BPH.

## 5. Conclusions

The study observed the influence of different depths of EA on BPH. At deep insertion during the treatment, greater improvement in the patients' urinary symptoms and quality of life was observed compared with patients that received shallow insertion EA. However, there was no significant difference between the two depths on the improvement of PVR and Qmax before and after the treatment. The results indicate that deep insertion has better therapeutic effects than shallow insertion for clinical application of the treatment for BPH via EA.

## Figures and Tables

**Table 1:** Baseline characteristics in two groups.

Item	DI group (*n* = 60)	SI group (*n* = 60)	*P* value
Age (years)	65.32 ± 8.62	64.02 ± 8.04	0.395
Course (years)	7.68 ± 4.34	8.30 ± 4.00	0.420
IPSS	22.08 ± 5.44	20.50 ± 5.40	0.112
QOL	4.50 (1.00)	4.00 (1.00)	0.361
PVR (ml)	30.20 (54.60)	40.25 (52.88)	0.572
Qmax (ml/s)	13.86 ± 4.79	12.30 ± 4.56	0.070

Notes: DI group (experimental group): EA with deep insertion about 60–75 mm. SI group (control group): EA with shallow insertion about 25–40 mm. IPSS: International Prostate Symptom Score; QOL: quality of life score; PVR: postvoid residual urine volume; Qmax: maximum urine flow rate. *P* value: between-group differences, *P* > 0.05 were not statistically significant.

**Table 2:** Comparison of the IPSS in each group pre- and posttreatment.

Item	DI group (*n* = 60)			SI group (*n* = 60)		
	Pre	Post	^a^ *P* value	Pre	Post	^b^ *P* value
Total IPSS	22.08 ± 5.44	11.57 ± 5.89	<0.001	20.50 ± 5.40	15.17 ± 5.51	<0.001
Incomplete emptying	4.28 ± 0.99	2.25 ± 1.73	<0.001	4.05 ± 1.33	2.43 ± 1.27	<0.001
Frequency	3.27 ± 1.38	1.55 ± 1.38	<0.001	3.28 ± 1.37	2.63 ± 1.50	0.004
Interruption	3.12 ± 1.91	1.87 ± 1.64	<0.001	2.95 ± 1.49	2.45 ± 1.87	0.081
Urgency	2.67 ± 1.84	1.08 ± 1.20	<0.001	2.23 ± 1.88	1.83 ± 1.36	0.139
Weak stream	3.33 ± 1.65	2.12 ± 1.85	<0.001	3.25 ± 1.59	2.47 ± 1.72	0.002
Hesitancy	2.18 ± 1.94	0.63 ± 0.97	<0.001	2.15 ± 1.84	1.20 ± 1.47	0.001
Nocturia	3.23 ± 1.67	2.07 ± 1.48	<0.001	2.58 ± 1.25	2.15 ± 1.07	0.021

Notes: DI group: EA with deep insertion about 60–75 mm. SI group: EA with shallow insertion about 25–40 mm. IPSS: International Prostate Symptom Score; IPSS contains 7 urinary factors including incomplete emptying, frequency, interruption, urgency, weak stream, hesitancy, and nocturia. ^a^*P* value: comparison between pre and post treatment in the DI group. ^b^*P* value: comparison between pre- and posttreatment in the SI group.

**Table 3:** Comparison of the pre-post-change of IPSS between the two groups.

Item (pre-post-change)	DI group (*n* = 60)	SI group (*n* = 60)	*P* value
Total IPSS	10.52 ± 6.71	5.33 ± 5.48	<0.001
Incomplete emptying	2.03 ± 1.94	1.62 ± 1.80	0.226
Frequency	1.72 ± 1.87	0.65 ± 1.66	0.001
Interruption	1.25 ± 2.18	0.50 ± 2.18	0.062
Urgency	1.58 ± 1.94	0.40 ± 2.07	0.002
Weak stream	1.22 ± 2.42	0.78 ± 1.87	0.274
Hesitancy	1.55 ± 2.20	0.95 ± 2.20	0.138
Nocturia	1.17 ± 1.85	0.43 ± 1.42	0.016

Notes: DI group: EA with deep insertion about 60–75 mm. SI group: EA with shallow insertion about 25–40 mm. IPSS: International Prostate Symptom Score; IPSS contains 7 urinary factors including incomplete emptying, frequency, interruption, urgency, weak stream, hesitancy, and nocturia. *P* value: differences in degree of change between two groups.

**Table 4:** Comparison of QOL, PVR, and Qmax in each group pre- and posttreatment.

Item	DI group (*n* = 60)			SI group (*n* = 60)		
	Pre	Post	^a^ *P* value	Pre	Post	^b^ *P* value
QOL	4.50 (1.00)	2.00 (2.00)	<0.001	4.00 (1.00)	3.00 (2.00)	<0.001
PVR (ml)	30.20 (54.60)	19.15 (21.30)	<0.001	40.25 (52.88)	15.00 (35.78)	<0.001
Qmax (ml/s)	13.86 ± 4.79	14.72 ± 4.81	0.117	12.30 ± 4.56	13.39 ± 4.31	0.017

Notes: DI group: EA with deep insertion about 60–75 mm. SI group: EA with shallow insertion about 25–40 mm. QOL: quality of life score; PVR: postvoid residual urine volume; Qmax: maximum urine flow rate. ^a^*P* value: comparison between pre- and posttreatment in the DI group. ^b^*P* value: comparison between pre and post treatment in the SI group.

**Table 5:** Comparison of the pre-post-change of QOL, PVR, and Qmax between the two groups.

Item (Pre-post-change)	DI group (*n* = 60)	SI group (*n* = 60)	*P* value
QOL	3.00 (1.00)	1.00 (2.00)	<0.001
PVR (ml)	13.4 (34.55)	17.10 (36.65)	0.709
Qmax (ml/s)	0.90 (3.45)	0.85 (3.22)	0.950

Notes: DI group: EA with deep insertion about 60–75 mm. SI group: EA with shallow insertion about 25–40 mm. QOL: quality of life score; PVR: postvoid residual urine volume; Qmax: maximum urine flow rate. *P* value: differences in degree of change between two groups.

**Table 6:** Clinical total effective rate in two groups.

Group	*n*	Highly effective	Effective	Ineffective	Effective rate (%)	*P* value
DI	60	15	26	19	68.33	<0.001
SI	60	1	22	37	38.33	

Notes: DI group: EA with deep insertion about 60–75 mm. SI group: EA with shallow insertion about 25–40 mm, 3 times a week for 4 weeks as a treatment course. *P* value: the differences of effective rate between two groups.

## Data Availability

The data used to support the findings of this study are available from the corresponding author upon request.
